# Case of Fracture and Subsequent Reunion of a Deciduous Superior Left Central Incisor Tooth

**Published:** 1872-09

**Authors:** 


### EDITORIAL DEPARTMENT.

*Case of Fracture and Subsequent Reunion of a Deciduous Superior Left Central Incisor Tooth.*—At the recent meeting of the the Southern Dental Association, Dr. W. Leigh Burton, of Richmond, Va., presented for examination a deciduous superior left central incisor which had been fractured at the neck by mechanical violence at the age of two years, and had subsequently united with the crown at a right angle with the root. This interesting specimen was accompanied by a paper, read before the Association by Dr. Burton, giving a history of the case, and which appears in full in the *Virginia Clinical Record* for July, Dr. Burton has kindly presented this interesting specimen to the Museum of the Baltimore College of Dental Surgey. The following history of the case will be interesting and instructive to our readers:

"The history of this somewhat remarkable case is substantially as follows: The child when bordering on two years of age had been, by her mother, put in her little bed, and was afterwards left in charge of a negro nurse. Some time about the middle of the night its parents were awakened by the cries of the child, and upon going to its assistance found its lips bleeding as if from a cut. As is usual in such cases, the nurse denied that any accident had happened, and insisted on it that the child was crying "jest so." In the morning, however, the lips were found to be much swollen and the gums so much inflamed and so painful to the touch, that a satisfactory examination of the injuries could not at the time be made; and though the swelling gradually subsided, the child almost continually complained of more or less pain. The parents were thoroughly convinced that the *alveolar process* had sustained some injury, but it was not until two years after the occurrence of the accident that they determined to place the child under the treatment of a dentist, and with this end in view, consulted the writer some time during the month of June of this year.

"Upon an examination being made it was found that the right central incisor had been fractured at the neck, and that the fang still remained in its socket; but in the case of the left, probing had to be resorted to in order to ascertain its condition, as it was nearly concealed by the gum with the exception of a small carious spot upon its labial surface, which gave it the appearance of a broken fang also. What appeared at first glance, however, to be a broken fang, proved to be the crown of the tooth which had evidently been driven inwards and fractured at the time the child sustained the injuries, and as it was nearly the time for the commencement of the second dentition, its extraction was at once decided on. Finding it impossible to apply the ordinary forceps in its removal, the elevator was resorted to, and when it had been dislodged, it was found that the tooth had beyond question been fractured, and that a perfect reunion had taken place at the point of contact of the fang and crown, and that the process of absorption was going on at the end of the fang as is usual in all deciduous teeth, as they are encroached upon by the permanent teeth. The union took place in the position in which the crown was discovered—nearly at angles to the fang, and now the question arises what agencies were concerned in producing this union?

"It is generally conceded that the development and formation of the teeth commences at a very early period of intra-uterine existence, so early in fact as within six weeks after conception, when small papillæ may be discovered occupying the positions intended for the future teeth. These gradually increase in size and assume the shape of the crowns of different teeth, and then commence to dentinify; the enamel is formed, and the process of solidification is completed before the tooth makes its appearance through the gums. The tooth then has lost its soft or glutinous character, is perfect in all its parts, and the enamel, dentine and cementum, having attained the requisite hardness, it is in a condition to be applied to the uses intended for it by nature.

"In attempting, therefore, to account for the union upon the hypothesis that *callus* had been thrown out, several objections at once confront this theory; for not only is the dentine of even a deciduous tooth much harder than ordinary bone, but the fracture of the fang would necessarily have snapped the nerve and blood vessels entering it, and thus being at once deprived of its vitality, it is questionable if this process could take place; nor is there that enlargement at the point of union which is characteristic of ordinary fractures of the bones. The theory then, of *callus* being thrown out, is, in the opinion of the writer, untenable, and the explanation must be found in a peculiar kind of exostosis usually developed on the points of the fangs of dead and diseased teeth; and in many cases this bony matter has accumulated to such an extent that it is impossible to remove the tooth without fracturing the alveolar process. The process of this formation is in many respects similar to that of the teeth. In the incipient stages a sac is formed, which in course of time becomes ossified and retains the shape and size of the original sac. It may therefore be concluded that sacs were formed simultaneously upon the fractured parts of the tooth, and being in such intimate connection the union occurred when ossification took place. In support of this theory it might be well to reiterate that exostosis is almost invariably developed upon the fangs of diseased teeth, and particularly upon those that have been fractured in the attempt to extract them; and it might be stated also, that when exostosis does not occur, the very reverse of this action takes place—the fang is wasted away by absorption until frequently it loses fully one-half of its original length, and the extraction of it rendered thereby comparatively easy."

